# A Fracture From a Physical Exam: A Case Report of Osteogenesis Imperfecta and the Use of Fassier-Duval Rods

**DOI:** 10.7759/cureus.37068

**Published:** 2023-04-03

**Authors:** Robert T Rella, Arcole S Brandon, Ian A Garrison, Patrick Young, Tyler C McDonald

**Affiliations:** 1 College of Medicine, University of South Alabama College of Medicine, Mobile, USA; 2 Orthopedic Surgery, University of South Alabama College of Medicine, Mobile, USA; 3 Pediatric Orthopedics, University of South Alabama College of Medicine, Mobile, USA

**Keywords:** fassier-duval, pediatric orthopedics, type 1 collagen, fracture, orthopedic surgery, osteogenesis imperfecta

## Abstract

Osteogenesis Imperfecta (OI) is a rare hereditary disorder that leads to fragile bone mineralization and is most often due to a genetic defect in type I collagen, the primary collagen subtype that comprises bone. Patients with OI suffer from a significant burden of fractures and bony deformities. It has been recognized in countries throughout the world and has a variable age and severity of presentation depending on the subtype of OI. Recognition of this disorder requires a high index of suspicion on the part of the clinician, as it can easily be mistaken for non-accidental trauma in children. The current approach to care for patients with this disorder comprises surgical care with intramedullary rod fixation, cyclic bisphosphonate therapy, and rehabilitation to maximize the patient's quality of life and function. This case report demonstrates the importance of considering OI in the differential diagnosis of a child presenting with recurrent fractures so that appropriate testing and treatment interventions can be implemented.

The case presented here is that of a male patient with osteogenesis imperfecta who suffered from recurrent long bone fractures, including his femurs bilaterally. His index fracture occurred after a visit to the pediatric ER for an unrelated issue, where his mother claimed that the boy demonstrated pain in his affected leg shortly after the visit. There was a delay in his diagnosis, and the patient suffered multiple fractures before undergoing the insertion of Fassier-Duval rods bilaterally into his femurs to prevent further injury.

## Introduction

OI is a rare hereditary disorder that leads to fragile bone mineralization and is most often due to a genetic defect in type I collagen, the primary collagen subtype that comprises bone [1]. Patients with OI suffer from a significant burden of fractures and bony deformities. It has been recognized in countries throughout the world and has a variable age and severity of presentation depending on the subtype of OI [1]. Recognition of this disorder requires a high index of suspicion on the part of the clinician, as it can easily be mistaken for non-accidental trauma in children. The current approach to care for patients with this disorder comprises surgical care with intramedullary rod fixation, cyclic bisphosphonate therapy, and rehabilitation to maximize the patient's quality of life and function. This case report demonstrates the importance of considering OI in the differential diagnosis of a child presenting with recurrent fractures so that appropriate testing and treatment interventions can be implemented.

Osteogenesis imperfecta is usually caused by a genetic mutation in type I collagen, and it can arise from over 30 different mutations and have a variable presentation ranging from mild bone abnormalities (OI type I) to mutations that are not compatible with life (type II) [2]. This patient presented with a substitution mutation of guanine to adenine in the COL1A1 gene located at the c.2829+1 site. This pathogenic variant abolishes the canonical splice donor site of intron 39 on COL1A1, which is likely to disrupt gene function. It has previously been identified in the literature multiple times and has been associated with types I and IV of OI [3,4]. It is believed that the majority of the cases of OI are type I and it is believed to be inherited in an overwhelmingly autosomal dominant fashion [2,4]. Type I collagen develops its strength from its heterotrimeric, triple-helical structure. It has two alpha 1 chains and one alpha 2 chains. Each collagen chain is first synthesized as a procollagen chain, and each procollagen chain has an N-terminal and a C-terminal. When the procollagen chains are assembled, the C-terminal will fold toward the N-terminal, and each side will flank the helical domain. In the helical domain are the glycine-x-y triplets, which are essential for the proper structure of type I collagen. The glycine side chain is necessary to fit in the internal helical space, but a glycine substitution causes delayed and, therefore, improper folding of the procollagen chains [2-4]. The inability to properly construct this type I collagen leads to the phenotypic manifestations of osteogenesis imperfecta.

## Case presentation

The case presented here is that of a male patient with osteogenesis imperfecta born at 33 weeks gestational age who was noted to have bilateral anterior bowing of his femurs and tibias at birth. He was born via cesarean section due to preterm labor and spent two weeks in the neonatal intensive care unit (NICU). His mother had a positive rapid plasma reagin (RPR) at birth, and orthopedics was consulted at three days of life due to his lower extremity deformity. It was postulated at the time that his bony deformity may be due to congenital syphilis, but no immediate intervention was required from a surgical standpoint at that time.

The patient followed up in the clinic with orthopedic surgery a few days after he left the NICU, and a skeletal survey at this time demonstrated bowing of the following: the bilateral tibias, the bilateral femurs, the bilateral radius bones, and the bilateral humerus bones. No acute fracture or dislocation was demonstrated at this time. A comparative genomic hybridization microarray was obtained at this time by his pediatric orthopedic surgeon due to clinical suspicion of OI and the bowing of the long bones seen on an X-ray. This assay was negative for osteogenesis imperfecta, proving later to be a false negative.

Family history is significant for the patient’s maternal great-grandmother having “thin bones, being bow-legged, and being particularly short”, according to the patient’s mother. She also stated that multiple members of her dad’s side of the family have “blue sclerae”. The patient’s maternal aunt is wheelchair-bound and started having “many fractures at the age of two”. There is no family history of consanguinity. The patient's father's family history is unknown, and the patient has two older brothers from the same father who are one and four years old with no known medical history. 

Initial fracture leading to a diagnosis

At eight weeks of age, the patient was initially brought to the emergency department due to nasal congestion, fussiness, and a decreased interest in oral intake for one day. The patient tested negative for the respiratory syncytial virus and influenza, and the patient’s mother was instructed to give the patient over-the-counter symptomatic treatments for his symptoms. After the patient returned home, he started screaming when his mother moved his legs to change his diaper. His left leg looked swollen the following morning, so she brought him back to the emergency department. His mother believed his left leg was injured when the Ortolani and Barlow maneuvers were performed on him by a clinician in the emergency department the previous night.

Upon physical exam, swelling on the proximal left thigh, pain with attempted passive movement of the left lower extremity at the hip, and lateral tibial bowing of the bilateral tibias were all immediately apparent. The patient was also noted to be in the 0.24 percentile for length. 

Laboratory studies at this time included a complete blood count, a comprehensive metabolic panel, and a prothrombin time, which were only significant for a leukocytosis of 20.49. Given the patient’s likely concurrent viral illness, the leukocytosis was not of particular concern at that time. 

**Figure 1:**
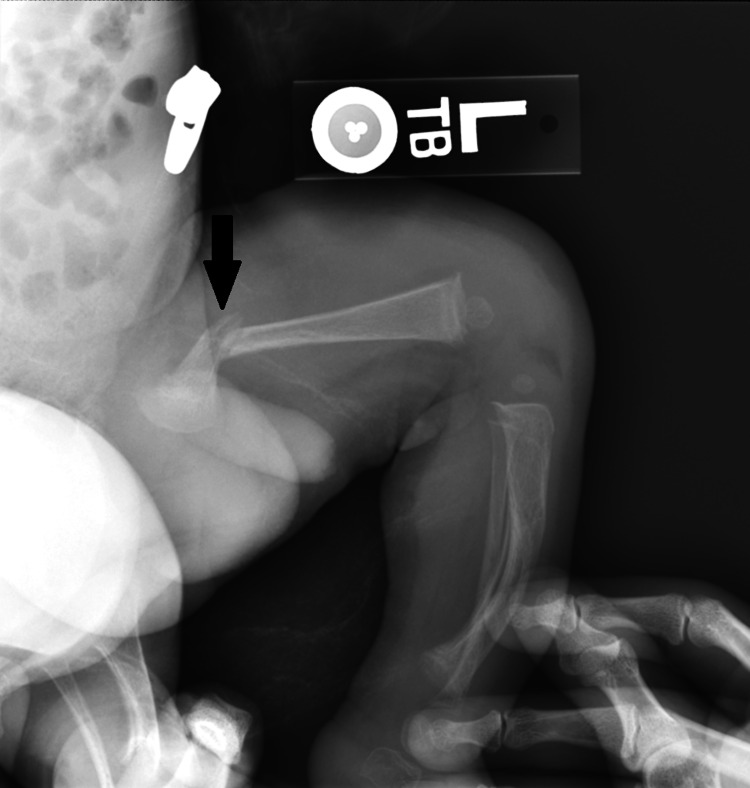
Radiographic view of the patient’s index fracture of the left femur, obtained after an unknown traumatic event to the left lower extremity. The black arrow denotes the fracture site.

Due to the noted swelling of the left femur, an X-ray was obtained that showed a diaphyseal fracture of the proximal left femur (Figure 1). Given the patient’s age and unknown mechanism of injury, a skeletal survey and head CT were obtained and showed bowing and periostitis at multiple sites, and multiple Wormian bones were seen at the confluence of the lambdoid and sagittal sutures and along the lambdoid sutures on the skull. At this point, child protective services were contacted due to a concern for non-accidental trauma, and he was admitted to the hospital for further evaluation.

The differential diagnosis at this time included congenital syphilis, a metabolic bone disorder, osteogenesis imperfecta, and non-accidental trauma, as these could all be plausible explanations for a bone fracture in the setting of an unexplained mechanism of injury. As stated earlier, the patient’s mother had a positive rapid plasma reagin at the time of delivery; however, both the patient and his mother tested negative with confirmatory treponemal testing at the time of delivery. The patient also received intramuscular penicillin after the delivery. Pediatric endocrinology was consulted at this time, and frontal bossing and a blue tint of the sclerae were noted as suspicion of OI began to rise. The patient underwent laboratory testing for metabolic bone disease, and the parathyroid hormone, vitamin D, calcium, phosphorus, and alkaline phosphatase were all within normal limits. Since there was a high suspicion of OI, child protective services declined involvement. The patient was discharged from the hospital in a Pavlik harness and followed up with orthopedic surgery one month later.

The patient then underwent genetic testing via a skeletal dysplasia panel from whole blood that demonstrated a pathologic variant in the COL1A1 gene, and this was consistent with a diagnosis of osteogenesis imperfecta. Approximately three months after his original fracture and diagnosis of OI, the patient began receiving pamidronate infusions every three months. The patient had a normal hearing test performed at age 21 months. Since this diagnosis, the patient has sustained the following fractures: a proximal humerus fracture (eight months of age), a right parietal bone fracture (nine months), a proximal to middle left femur fracture (three years), a right subtrochanteric femur fracture (three years), and a distal right femoral fracture (three-and-a-half years). From an orthopedic perspective, these fractures were treated with closed reduction spica cast application, closed-reduction, percutaneous pinning, femoral intramedullary nail insertion, and finally, insertion of bilateral femoral Fassier-Duval (FD) rods. 

Fassier-Duval rod insertion at 44 months of age

**Figure 2:**
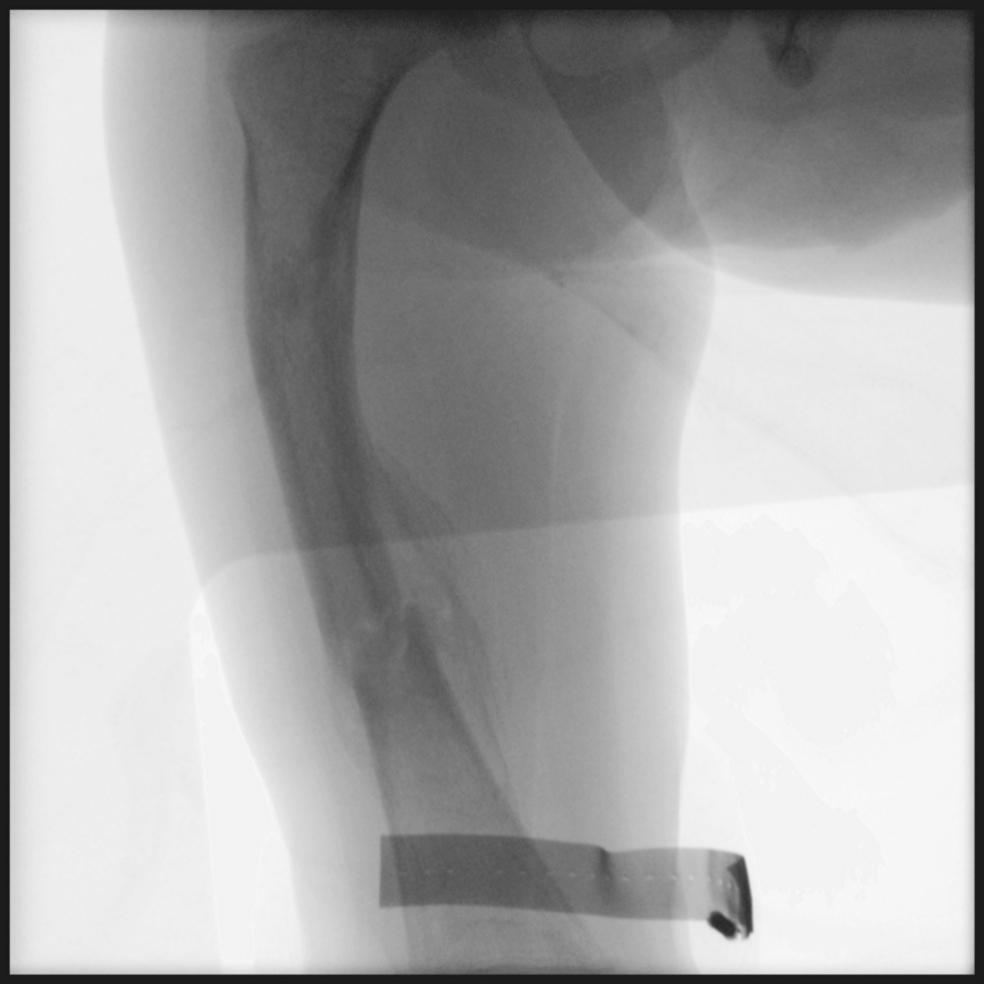
Intraoperative anteroposterior view of the right femur immediately after hardware removal in preparation for osteotomy creation and Fassier-Duval rod insertion.

When the patient was finally brought to the operating room, the procedure began, starting on his right thigh. First, the previously placed k-wires were removed from the distal femur without issue (Figure 2). Next, a guide wire was placed in the proximal femur and advanced to the subtrochanteric region, where the first bowing deformity was encountered. Then, the proximal segment was cannulated, and a guide wire with a small distal bend was advanced into the proximal segment, and an awl was used to make a hole in the femur at this level, creating a stress riser. The leg was then bent with the intent to fracture the femur at this location in a controlled fashion, creating an osteotomy. The same procedure was repeated at the second area of deformity in the mid-femur. The guide wire was then advanced and placed in the center of the medullary canal on both the anteroposterior and lateral views (center-center).

After this, the male component was inserted anterogradely into the medullary canal after it was cannulated, with care taken not to cross the distal femoral physis. The female component was cut to size and inserted anterogradely over the male component and secured into the trochanter until the top was level with the center of the femoral head. After final imaging, the right leg was irrigated and closed before the left leg was prepared for surgery.

**Figure 3:**
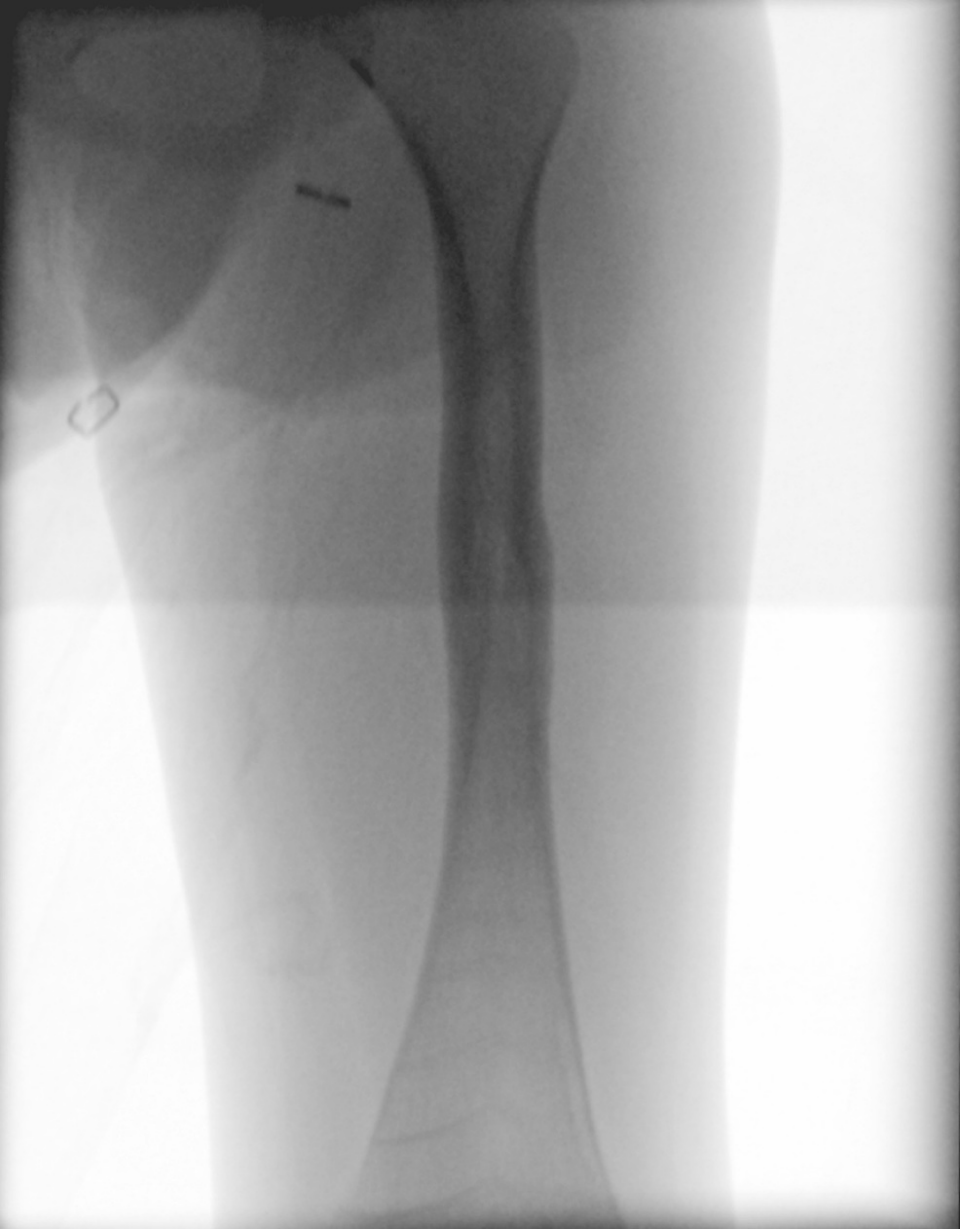
Intraoperative anteroposterior fluoroscopic view of the left femur prior to ostomy creation and insertion of the Fassier-Duval rod.

Because the left femur had a large apex-anterior bowing deformity in the proximal diaphysis, an open dissection was performed to access the femur and perform an ostomy, as opposed to the closed ostomies that were made in the right femur (Figure 3). After the osteotomy was performed and the guidewire was found to be center-center, the left femur was reamed carefully as before. Then the male and female components of the Fassier-Duval implant were inserted as previously described. A mixture of a demineralized bone matrix with bone chips and powder was made and placed around the open osteotomy site, and the incisions on the left leg were irrigated and closed.

Postoperative course

**Figure 4:**
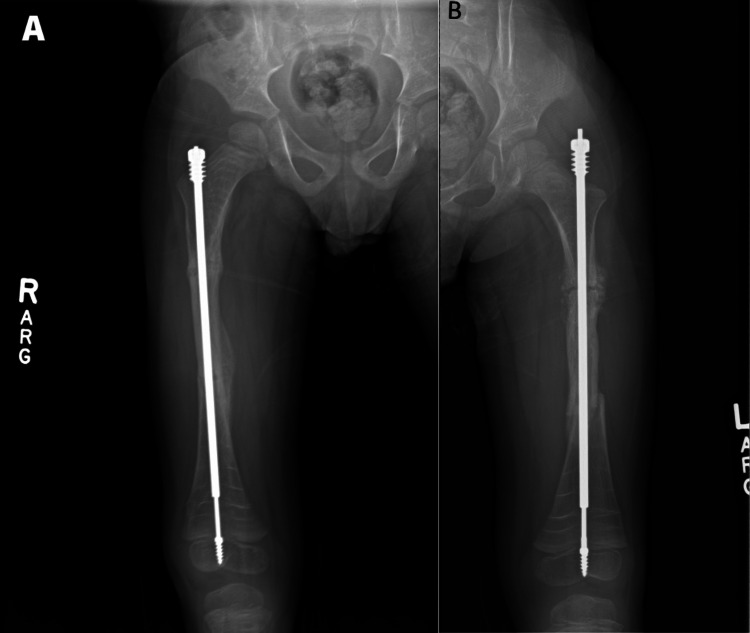
Postoperative radiographs demonstrating proper alignment of the implants and appropriate healing eight weeks after the operation.

The patient was placed in a double-leg spica cast, holding the hips and knees in flexion with slight external rotation of the hips. He was taken to the post-anesthesia care unit (PACU) in stable condition and recovered from the surgery during his hospital stay uneventfully, returning home on post-op day (POD) two. The patient has recovered well, with his mother reporting that he has been very active at home, rolling and kneeling frequently before he began weight bearing as tolerated after his eight-week follow-up visit. Postoperative radiographs demonstrated healing of his osteotomies and that the female component of the left FD rod had backed out slightly, but that the rod was holding alignment and could be observed clinically over follow-up (Figure 4). At that time, there were no further complaints.

## Discussion

This patient presented with multiple fractures after minimal trauma, which is a hallmark of the disease, but he also presented with other findings associated with OI, such as blue sclera, a short stature, frontal bossing, and a strong family history suggestive of the disorder. All of these findings, along with the confirmatory genetic testing, are diagnostic of osteogenesis imperfecta, but other important findings correlate with OI. Another classic finding in osteogenesis imperfecta is that of dentinogenesis imperfecta, in which the teeth are noted to be discolored and can undergo easy destruction [5]. Other findings can include scoliosis, aortic and mitral regurgitation, hearing loss, functional pulmonary impairment, and skull base abnormalities, such as basilar impression and basilar invagination [2]. Knowledge of the associated comorbidities is essential for treating clinicians due to the need for frequent follow-ups with different specialties. A multidisciplinary team is required for patients with OI, including primary care physicians, orthopedic surgeons, cardiologists, dentists, physical therapists, and audiologists, due to the aforementioned comorbidities associated with the disorder. Furthermore, these patients require a low threshold for neurological imaging if they present with new central nervous system symptoms, such as nystagmus or ataxia, due to the concern for basilar abnormalities. 

The FD nail is a medical device developed for the treatment of OI. The FD nail is designed to stabilize the long bones of the extremities and promote bone growth, which can improve bone strength and reduce the risk of fractures in patients with OI. Although elongating rods have been in use for about two decades, estimates of the clinical outcomes vary, with complication rates reported as high as 61% and reoperation rates of 78%. This reoperation rate has dropped with each successive generation of elongating rods; however, it has been reported that there is about a 9% complication rate per rod per follow-up year associated with these elongating rods [6]. A case series of 19 patients with OI found a complication rate of 44.8% over four years of follow-up, with female component migration, as seen in our patient, accounting for a quarter of all complications [7]. As available options for elongating rods continue to improve, these high rates of complications and reoperations will likely continue to decline.

Revision surgery is common among patients with FD nails. Hung et al., in their study of seven patients with OI, found that revision surgery was required in 11 of the 20 limbs containing FD rods. The main reasons for revision surgery were migration of the male or female component, refracture of the limb or nail bending, and delayed union. Common reasons for implant failure in the femur include bending and female component migration. The average interval between previous FD nail insertion and revision surgery was 2.4 years [8]. Taking this into consideration, we can reasonably conclude that the female component migration in the left femur experienced by the patient in this study is not an unforeseen complication that may require reoperation in the future.

Ambulation is improved following FD nail insertion and can prevent repeated cast application, osteopenia, and disuse wasting. In this manner, FD rods improve mobility potential and prevent deterioration in the quality of the long bones [9]. Compared to static implants (Rush rods, flexible nails, and Steinmann pins), FD nails have a reported 13.2 times lower hazard of implant failure. Interestingly, it was found that females have a 4.8 times greater hazard of implant failure than their male counterparts. Those with FD rods have been found to have lower rates of surgery than those with static implants [10]. It is clear from the literature that FD nailing is an imperfect solution to a systematic disease, and the standard of surgical care stands to benefit from continued improvements in the techniques and implants available to patients.

Living with osteogenesis imperfecta can potentially come at a great cost to the patient. Children are required to take great caution in their daily lives, and proper protective equipment, such as a helmet while the patient is riding a bicycle, is essential, as even injuries that most children would consider minor and insignificant can have serious consequences for children with OI. The current standard of care is symptomatic treatment with bisphosphonates to inhibit the bone resorptive activity of osteoclasts [11]. Dr. Glorieux demonstrated multiple positive outcomes with this treatment regimen, including increasing both the cortical width and amount of cancellous bone in the measured bones while reducing indicators of bone remodeling [12]. Another pharmacologic option being explored is that of the monoclonal antibody to RANKL, denosumab, with promising early results in children with OI. Other pharmacologic therapies being studied for the treatment of OI include PTH analogs, sclerostin inhibitory antibodies, and transforming growth factor beta inhibitors, but the standard of care for patients with OI remains bisphosphonates [13].

Another symptomatic approach to treatment is that of the aforementioned FD rods. There is currently no definitive treatment for OI, but stem cell transplants have previously been carried out with promising results, yet too few patients have been treated in this manner to endorse it as a standard of care [11]. Previous efforts to silence the mutated gene have been partially successful, but the true definitive treatment could come in the form of gene editing with the CRISPRs/Cas9 system. This has not yet been tried in patients with OI, but it holds a promising future in terms of treating genetically mutated Mendelian disorders. 

The differential diagnosis for osteogenesis imperfecta is broad and includes metabolic disorders, such as rickets; congenital disorders, such as neonatal syphilis; but most importantly, it includes non-accidental trauma. One of the hallmarks of non-accidental trauma is a story from a patient’s caregiver that does not match up with the clinical presentation of the patient. For instance, when this patient presented with his initial fracture, his caregiver stated it was due to the emergency department provider being too rough with the child during the physical examination, yet this story did not match with the femoral fracture that was seen on the radiological imaging. Clinicians must always rule out non-accidental trauma, which is usually done with a skeletal survey, a fundoscopic eye exam looking for retinal hemorrhages, a good dermatological exam documenting any bruising, and a non-contrast head CT to look for any signs of a cerebral hemorrhage. It is always necessary to contact the local child protective services agency anytime that non-accidental trauma is suspected, but it is also important to examine the patient for other pathologies that could explain the fractures, such as osteogenesis imperfecta. Obtaining a good history, including that of any previous fractures in the patient and a family history of repeated fractures, and performing a thorough physical examination are both appropriate ways to evaluate for causes of the fracture other than non-accidental trauma. Both non-accidental trauma and osteogenesis imperfecta can cause many significant problems in the life of a young patient, but the treatment is very different for these two causes of repeated bone fractures, which is why the underlying cause must be differentiated as expeditiously as possible.

## Conclusions

This case demonstrates the importance of having a broad differential diagnosis that includes osteogenesis imperfecta for the presentation of a pediatric patient with a fracture that is inconsistent with the stated history. It also illustrates the absolute necessity of ruling out non-accidental trauma in patients with this presentation. While medical management is a mainstay of therapy for patients with OI, surgical intervention offers significant benefits to patients with regards to improvements in quality of life and reduced rates of fracture and re-injury. It is through the effective synergy of medical, surgical, dental, social, and therapeutic services in a multidisciplinary team that optimal care for patients suffering from OI can be most thoroughly provided.
